# Identification of Bacteriophage Virion Proteins Using Multinomial Naïve Bayes with g-Gap Feature Tree

**DOI:** 10.3390/ijms19061779

**Published:** 2018-06-15

**Authors:** Yanyuan Pan, Hui Gao, Hao Lin, Zhen Liu, Lixia Tang, Songtao Li

**Affiliations:** 1 School of Computer Science and Engineering, Center for Informational Biology, University of Electronic Science and Technology of China, Chengdu 610054, China; panyy0422@163.com (Y.P.); quake@uestc.edu.cn (Z.L.); lst1994824@163.com (S.L.); 2 Key Laboratory for Neuro-Information of Ministry of Education, School of Life Science and Technology, Center for Informational Biology, University of Electronic Science and Technology of China, Chengdu 610054, China; l.x.tang@163.com

**Keywords:** bacteriophage virion proteins, g-gap peptides, ANOVA, Multinomial Naïve Bayes

## Abstract

Bacteriophages, which are tremendously important to the ecology and evolution of bacteria, play a key role in the development of genetic engineering. Bacteriophage virion proteins are essential materials of the infectious viral particles and in charge of several of biological functions. The correct identification of bacteriophage virion proteins is of great importance for understanding both life at the molecular level and genetic evolution. However, few computational methods are available for identifying bacteriophage virion proteins. In this paper, we proposed a new method to predict bacteriophage virion proteins using a Multinomial Naïve Bayes classification model based on discrete feature generated from the g-gap feature tree. The accuracy of the proposed model reaches 98.37% with MCC of 96.27% in 10-fold cross-validation. This result suggests that the proposed method can be a useful approach in identifying bacteriophage virion proteins from sequence information. For the convenience of experimental scientists, a web server (PhagePred) that implements the proposed predictor is available, which can be freely accessed on the Internet.

## 1. Introduction

A bacteriophage is a virus that is inhabited in bacteria and consists of DNA, RNA, viral proteins, and packaging proteins. Bacteriophages infect bacteria and then result in its lysis [1]. One bacteriophage could infect one or more species of bacteria [2] and almost 20% of bacteria are lysed through bacteriophages infection each day. Bacteriophages have been shown to encode a range of functional proteins that influence their bacterial cells or even the host in which the bacterium lives [3]. Bacteriophages actually play an important role in host bacteria genome evolution.

Bacteriophages also play an important role in the research of bacterial infections, especially bacterial drug resistant infections [4,5,6]. Bacteriophages infect bacteria by binding to the specific receptors on the surface of the bacterial cell. The specific receptors needed in the interactions protect the unrelated pathogens and infect the target accurately. As fundamental materials of the infectious viral particles, bacteriophage proteins have important biological functions in the interaction between bacteriophage and host bacterial cell. Bacteriophage proteins include structural (virion) proteins and non-structural (non-virion) proteins. The bacteriophage virion proteins participate directly in the evolutionary contest between themselves and their hosts. Bacteriophage non-virion proteins, also play important roles in bacteriophage replication, transcription and polyprotein processing. Since 1959, at least 5568 bacteriophages have been discovered [7]. New bacteriophages are continually found because of the unremitting efforts of scientists. Due to the rapid advances in genomic and proteomic research in recent years, tremendous amounts of DNA and protein sequences have accumulated in databases. The traditional techniques for protein research, such as Mass spectrometry, have been proved correct but inefficient. Hence, it is highly desirable for computational biologists to develop a practical approach that efficiently extracts relevant biological information from sequences to identify the bacteriophage virion proteins.

Li et al. developed a system called SynFPS [8] to perform gene function prediction over completed genomes. SynFPS clustered the genomes by their resemblance in gene distribution, and then, for each individual group, the data is extracted and used to train a Support Vector Machine for gene function predictions. In the work of Seguritan et al. [9], the frequency of amino acids extracted from the protein sequence was used as input to train the Artificial Neural Networks (ANN) and subsequently, protein isoelectric points were fed into ANNs to classify specialized families of proteins. Their aim was to predict bacteriophage structural protein sequences by ANNs. Feng et al. proposed a Naïve Bayes based method to identify bacteriophage virion proteins which achieved an overall accuracy of 79.15% in jackknife cross-validation [10]. Subsequently, they used the analysis of variance (ANOVA) as the feature importance criterion to select the g-gap dipeptide as the important feature for the bacteriophage prediction and then applied support vector machine (SVM) classifier to identify bacteriophage virion proteins [11]. A list of 160 feature set was used to encode each protein sequence of which they obtained an accuracy of 85.02% in jackknife cross-validation. Zhang et al. used an ensemble method for bacteriophage virion protein prediction from bacteriophage protein sequences which was put forward with hybrid feature spaces incorporating CTD, bi-profile Bayes, PseAAC and PSSM [12]. Their method achieved an accuracy of 85.30% with 10-fold cross-validation. Shin et al. [13] described an SVM-based PVP predictor called PVP-SVM which was trained with the same data. The randomforest algorithm was employed to select the optimal features from a large set that included amino acid composition, dipeptide composition, atomic composition, physicochemical properties, and chain-transition-distribution. PVP-SVM achieved an accuracy of 86.97% in jackknife cross-validation. The bacteriophage prediction is worthy of further investigating because the prediction performance is still far from satisfactory.

**Figure 1:**
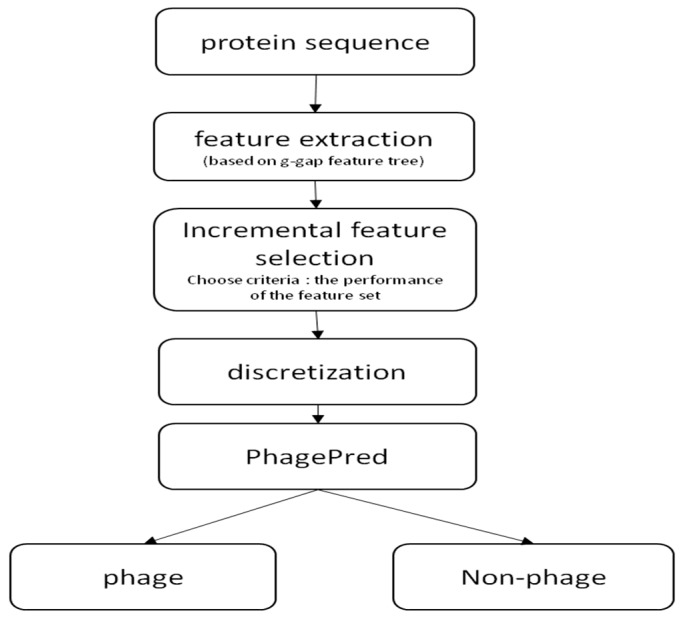
The computational framework of the PhagePred.

In previous work, a g-gap dipeptide composition describing the long-range correlations between two residues was proposed and had demonstrated its effectiveness in the realm of protein identifications [14,15,16]. However, in most cases, the functional motifs of proteins are often constituted with more than two discontinuous residues. Such useful information unfortunately could not be successfully extracted by the g-gap dipeptide composition. To overcome this limitation, we proposed a novel method to identify bacteriophage proteins using Multinomial Naïve Bayes (MNB) with g-gap feature tree. A summary of the computational framework of our method is illustrated in Figure 1. We firstly constructed a g-gap feature tree to pick out a number of informative features from protein sequence information. We then used the discretization techniques to transform the top K optimal features into qualitative data, obtained by ANOVA. The protein sequence of each sample in the bacteriophage dataset was then transformed into a K-dimensional discrete feature vector. Finally, we performed Multinomial Naïve Bayes classification on the discrete feature vectors of all samples to establish the prediction model. Results from 10-fold cross-validation test demonstrate that the proposed model achieves a remarkable improvement in overall accuracy. Based on this prediction model, a free online server called PhagePred was built to provide a useful tool for identifying bacteriophage proteins.

## 2. Results

### 2.1. Comparison of Discrete Feature Vector in Different Dimensions

**Figure 2:**
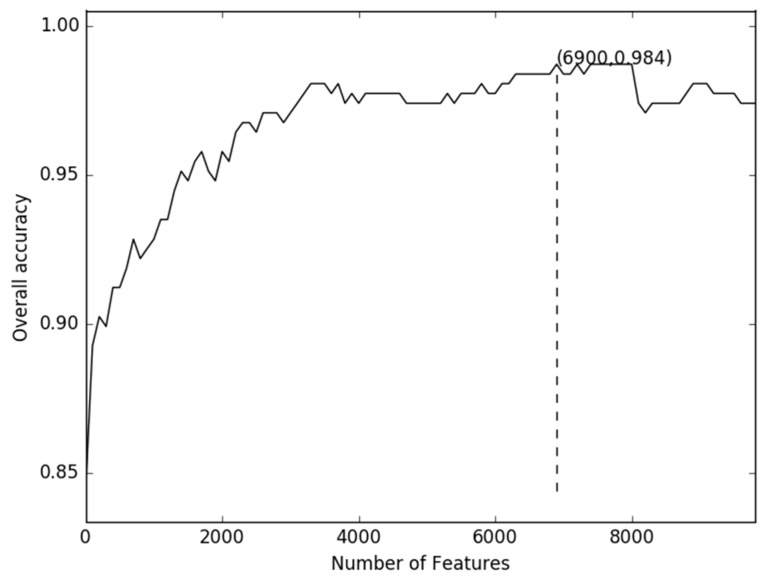
The incremental feature selection curves for the values of accuracy against the discrete feature vector.

For very high dimensional data (49,220 dimensions in this paper), using dimensionality reduction techniques like Principal Component Analysis (PCA), Latent Dirichlet Allocation (LDA) or Probabilistic Latent Semantic Analysis (PLSA), not only alter the original representation of the variables but they are also computationally expensive [17]. A less expensive approach to dimensionality reduction is feature selection, which reduces the number of features by selecting a subset from the original feature set based on some chosen criteria. In particular, feature selection by ANOVA selects the top features that have the highest differences between the means of two groups to remove redundant or irrelevant features and improve classifiers’ accuracy. In order to find a minimum set of features that achieves maximum classification performance (for a given set of classifiers and classification performance metrics), the incremental feature selection was used to determine the optimal feature set. The accuracy of the data reached its peak (98.37%) when the top ranked 6900 features were used. Figure 2 shows a 10-fold cross-validation accuracy with different numbers of features.

### 2.2. Comparison of g-Gap Features with Others

**Figure 3:**
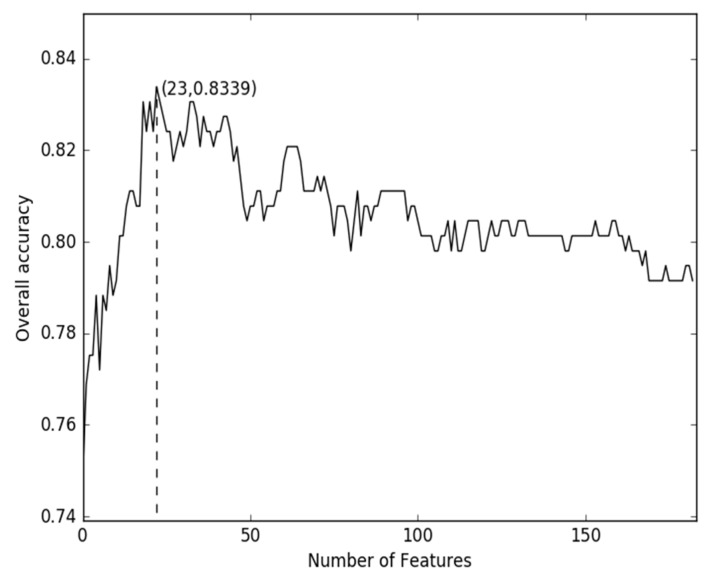
The incremental feature selection curve for the values of accuracy against the combination subsets.

Liu et al. [18] generated various feature vectors from the protein sequence that can be grouped into three categories. The first category is the occurrence frequencies of k neighboring amino acids (k-mer). The second category is autocorrelation, reflecting three different manners (auto covariance, cross covariance, auto-cross covariance) in counting the correlations along a protein chain via amino acid physicochemical properties. The third category is pseudo amino acid composition (PseAAC) for incorporating the global or long-range sequence order information of protein sequences into feature vectors via the physicochemical properties of constituent amino acids. Using the proposed model with the feature mentioned above, the performance was depicted as shown in Figure 3. From Figure 3, the best recorded accuracy of the combined-feature is 83.39% which is lower than our method.

### 2.3. Comparison with Different Classifiers

Here we investigate whether or not the Multinomial Naïve Bayes classifier with discrete features can significantly improve the performance of bacteriophage virion protein prediction compared with other classifiers. The proposed method was compared with several state-of-the-art classifiers such as xgboost, Random Forest, Adaboost classifier over Classification and Regression Trees (CART) and SVM. The performance comparison of these different classifiers was obtained by 10-fold cross-validation (as shown in Table 1). We compared the performance of Multinomial Naïve Bayes classifier with the other classifiers using the same feature subset of size 6900. All models were tested on the dataset containing 99 positive and 208 negative sequences.

**Figure 4:**
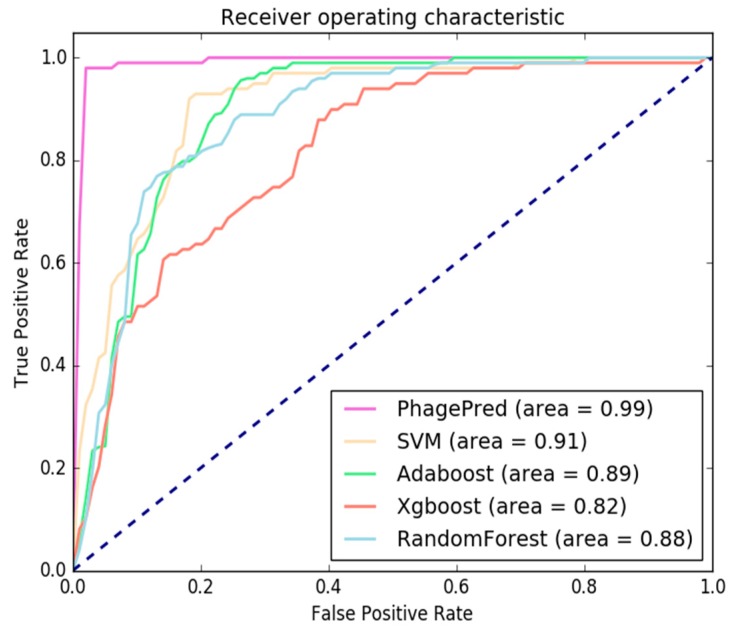
The receiver operating characteristic (ROC) curves calculated from the 10-fold cross-validation of the five different classifiers.

As shown in Table 1, our method outperforms all other four classifiers in terms of all the specified performance measures. Compared with Random Forest, the Sp value of MNB is improved from 97.60% to 99.04%. The accuracy of MNB is raised by 13.03% compared with SVM. In order to compare the performance of our model with other models more intuitively, we present the ROC curves for all five models in Figure 4. The ROC Curves Chart in Figure 4 presents the true positive and false positive rate on the test data at different thresholds for the classifiers using the top 6900 features. The area under receiver operating characteristic curve (AUC) for PhagePred is 0.99 and the other classifiers are 0.91, 0.89, 0.82 and 0.88, respectively. The AUC for PhagePred is greater than the others, suggesting that PhagePred may provide a better predictive method for bacteriophage virion proteins.

### 2.4. Comparison with Existing Methods

In general, if a prediction model is developed using a training dataset that contains highly homologous sequences, it may overestimate the prediction accuracy. In this regard, a lower homology (<40% sequence identity) sequence dataset was used to develop prediction models in [10,11,13]. Zhang et al. developed their model using a highly homologous sequence dataset (<80% sequence identity) [12]. In this paper, we compared the performance of our method with Naïve Bayes [10], SVM [11], and PVP-SVM [13].

In previous work, Feng et al. used Naïve Bayes as classifier to predict bacteriophage virion proteins [10]. A method named PseAAC was proposed to represent the protein sequences and correlation-based feature selection combined with best-first search strategy was adopted to remove irrelevant features. Feng et al.’s method achieved an accuracy of 79.15% in the jackknife test. With g-gap dipeptide compositions as the features, ANOVA as the feature selection method and SVM as the classifier, Ding et al. produced a maximum accuracy of 85.02% in jackknife cross-validation [11]. PVP-SVM in [13] achieved an accuracy of 86.97% during jackknife cross-validation. By selecting the top 6900 F-score features as the input to our model, we achieve a 98.05% accuracy score in the jackknife test. The performances of the methods mentioned above are shown in Table 2. As shown in Table 2, the accuracy of our model increased nearly 7% from the best method with the highest Acc, besides, the values of Sn and Sp were increased by 20% and 5%, respectively. Although we use a larger feature set, due to the simplicity of the Naïve Bayes model, the execution time of the method is within an acceptable range. When the size of feature set is 160 (the same as [11]), the Acc of PhagePred is 86.97%, the Sn and Sp is 89.89% and 85.58%, respectively. It shows that PhagePred outperforms the existing methods even if a low dimensional feature vector is used. The experimental results mentioned above demonstrate that our model outperforms all other previous models on this dataset.

## 3. Discussion

Even though a number of computational methods have been used to predict bacteriophage virion proteins, the performance of previous prediction models can still be enhanced. The functional motifs are the signature of some protein family and are often constituted with two or three discontinuous residues. In this work, we consider many more features according to the patterns extracted from the g-gap tree. The maximum depth of g-gap feature tree is actually limited by the memory and the processing time of the computer. The g-gap feature tree stops expanding when all the patterns generated in the leaf nodes contain more than three amino acids residues or three gaps. After we rule out all the sequence patterns that either begin or end with a gap, we obtain 10 patterns from the tree, which corresponds to a big set of around 49,220 features. In comparison with other feature extraction methods, the big set of features we extracted turn out to have a better discriminative capability according to the result of the experiment (see Figure 2). 

The Multinomial Naïve Bayes classifier assumes that the conditional probabilities of the independent variables are statistically independent. In this work, the Multinomial Naïve Bayes classifier is applied to predict the bacteriophage virion proteins, and it is capable of dealing with a large amount of these features without causing the problem of overfitting. From Table 1, we can see that the performance of our model outperforms all other classifiers.

## 4. Materials and Methods

### 4.1. Benchmark Dataset

The original dataset, described by [10], was collected from the UniProt [19]. To guarantee the quality of the benchmark dataset, they excluded the protein sequence which contained ambiguous residues (such as ‘X’, ‘B’ and ‘Z’). Secondly, if a sequence was a fragment of other proteins, it was excluded from the dataset. Thirdly, to avoid any similarity bias which would result in an overestimation of predicted results, the CD-HIT program [20] was used to remove highly similar sequences by setting the cutoff of sequence identity at 40%. The remaining 99 bacteriophage virion proteins formed the final positive dataset and 208 non-virion bacteriophage formed the negative dataset.

### 4.2. g-Gap Feature Tree

In this paper, we build a binary tree named g-gap feature tree to describe as many functional motifs of proteins as possible. For each node of the g-gap feature tree, its value corresponds to a set of g-gap features, which can be represented in the same form as a sequence pattern. 

**Figure 5:**
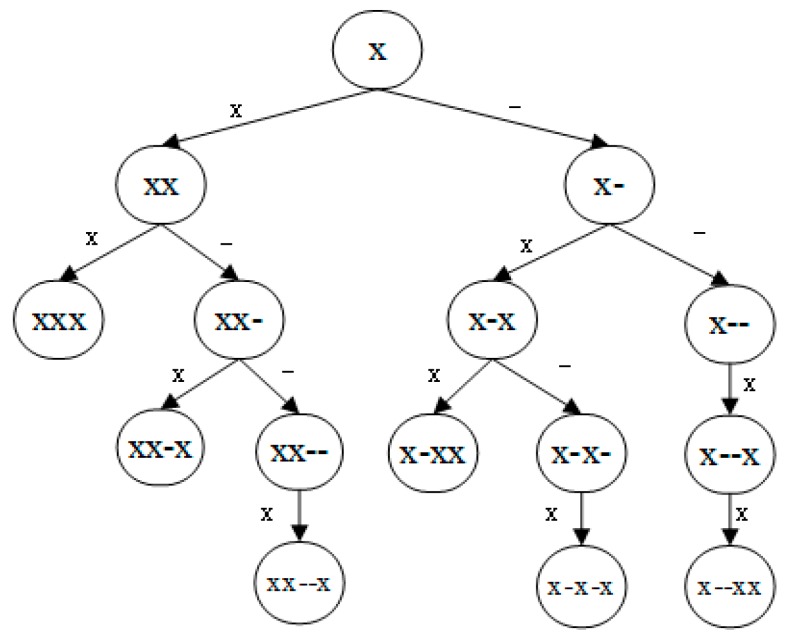
The g-gap feature tree.

To define a sequence pattern, we use symbol ‘*x*’ to denote one of 20 amino acids (i.e., x∈{A,C,D,E,F,G,H,I,K,L,M,N,P,Q,R,S,T,V,W,Y}), and symbol ‘−’ to denote one gap between two discontinuous amino acids. The pattern xx denotes a set of all 20×20=400 dipeptides (i.e., A,AC,…,YY), the pattern x−x denotes a set of all 20×20=400 one-gap dipeptides (i.e., A−A,A−C,…,Y−Y), and so on. To build the g-gap feature tree as depicted in Figure 5, we start from a tree with only one node, which is both the root node and the leaf node. This single node of the tree corresponds to a set of features that are in the form of pattern *x*. We repeat the following process to expand the tree from every leaf node to its left and right until the patterns of all leaf nodes contain more than three amino acids residues or three gaps. When a leaf node is expanded to its left and right, the pattern of the new left child is the concatenation of the pattern of its parent node with ‘*x*’, and the pattern of the new right child is the concatenation of the pattern of its parent node with ‘−’. After a leaf node expands to its left and right, it becomes an internal node of the tree, and its two new children become new leaf nodes of the tree. The expanding process of the tree terminates when all the leaf nodes stop expanding. We rule out all the sequence patterns that either begin or end with a gap. So, all the patterns we generated from the g-gap feature tree are: x,xx,xxx,x−x,x−xx,xx−x,x−−x,x−−xx,xx−−x,x−x−x.

### 4.3. Discrete Feature Vector and Classifier

Based on protein sequence information, we first compute the frequency of every feature corresponding to a node in the g-gap feature tree. In this way we transform the sequence into quantitative features. Then, we calculate the F-score by ANOVA to evaluate the discriminative capability of all the features in the tree. After that, we sort the features by ascending order of their F-scores and choose the first K features as the result of feature reduction. For the chosen K features, we discretize these continuous valued data into discrete data by the discretization process. In that way, we can convert each sequence sample in the bacteriophage dataset into a K dimensional discrete feature vector (DFV), and later feed the discrete feature vectors of all the samples into the Multinomial Naïve Bayes classifier for identifying the bacteriophage virion protein.

#### 4.3.1. Transformation

The density of every feature corresponding to a node in the g-gap feature tree was used to transform the protein sequences into quantitative features. For convenience of discussion, we denote a query protein sequence with L amino acid residues as

(1) $$p=s_{1}s_{2}s_{3}\ldots s_{L}$$

where $s_{1}$ represents the residue at position 1, $s_{2}$ represents the residue at position 2, and so forth. In the sequence **p**, each residue $s_{i}$ (1≤i≤L) belongs to a set of 20 different amino acids. We compute the density [21] of feature *f* ($j_{th}$ feature in the feature set) based on local protein chain description [22] of **p** ($i_{th}$ sample in the dataset) as follows:

(2) $$f_{i,j}=density_{p,f_{i,j}}=\frac{1}{L^{\prime }}\overset{L^{\prime }}{\underset{k=1}{\sum }}I\left(p_{k}\right))$$

where $f_{i,j}$ is the frequency of $i_{th}$ feature of the $j_{th}$ sample; $L^{\prime }$ denotes the total number of the sliding subsequence concerned; $p_{k}$ is the $k_{th}$ sliding subsequence in sequence **p**, and

(3) $$I\left(p_{k}\right)=\{\begin{array}{cc} 0, & if\;p_{k}==the\;feature\;concerned.\\ 1, & otherwise. \end{array}$$

#### 4.3.2. ANOVA

The one-way ANOVA test is based on F statistic. The higher the F ratio value, the better the discriminative capability of the feature [23]. F ratio is calculated for each of the feature *f* ($j_{th}$ in the feature set) in this study as follows:

(4) $$F_{j}=\frac{MS_{between}}{MS_{within}}=\frac{SS_{between}}{df_{between}}/\frac{SS_{within}}{df_{within}}=\frac{\sum_{\mu =1}^{M}n_{\mu }\bar{(f\left(\mu \right)}-\bar{f}{)}^{2}}{M-1}/\frac{\sum_{\mu =1}^{M}\sum_{i=1}^{N}{\left(f_{i,j}\left(\mu \right)-\bar{f\left(\mu \right)}\right)}^{2}}{N-M}$$

where *M* and *N* denotes the number of classes and the total number of samples, respectively. $\bar{f\left(\mu \right)}$ is the mean of considered feature for the $\mu_{th}$ class, $\bar{f}$ is the grand mean of feature *f* considering all classes in all samples, $f_{i,j}\left(\mu \right)$ represents the value of feature *f* of the $i_{th}$ sample of the $j_{th}$ feature on the $\mu_{th}$ class and $n_{\mu }$ is the number of samples for the $\mu_{th}$ class.

#### 4.3.3. Discretization

Discretization is a process that transforms quantitative data into qualitative data. A many to one mapping function is created so that each value of the original quantitative attribute is mapped onto a value of the new qualitative attribute. Discretization is considered as a data reduction mechanism since it diminishes data from a large domain of continuous data values to a subset of categorical values. Compared with continuous attributes, discretization is easy to handle and closer to knowledge level representation [24]. In this paper, an unsupervised discretization method was used to regroup the density of amino acids into three classes. Namely, class 0 represents low concentration of amino acids, 1 and 2 denote the mid and high concentration respectively. In order to split the continuous data into the non-overlapping domain, we first sort the data by either descending or ascending order, and then find the optimal cut-off point for the specific feature. Precisely, the cut-off points for each attribute must be considered respectively. Next, we explain these discretize processes in detail. For the discretization of feature $f=f_{i,j}$, we first sort the continuous values of the feature *f* by ascending order and then evaluate a cut-off point for splitting. The samples whose density was 0 were mapped into the symbol 0, and those were excluded from the samples we used to find the cut out point. The median (*m*) of non-zero samples’ value was used as the optimal cut-off point, through which we obtain the two intervals for the feature f:(0,m] and (m,∞]. We then, split the continuous values to the discrete symbol in accordance with the divided intervals. For example, if $f_{i,j}\in \left(0,m\right]$, the $j_{th}$ feature of $i_{th}$ sample is labeled with symbol 1; if $f_{i,j}\in \left(m,\infty \right]$, the $j_{th}$ feature of $i_{th}$ sample is labeled with symbol 2. In this way, the original features’ value will be mapped into the discrete value 0, 1, 2 as expected.

#### 4.3.4. Multinomial Naïve Bayes

The Naïve Bayes Classifier [25] technique is based on the so-called Bayesian theorem and is particularly suited when the dimensionality of the inputs is high. In order to reduce the complexity of this high dimensionality, the Naïve Bayes classifier assumes that the conditional probabilities of the independent variables are statistically independent. Despite its simplicity, Naïve Bayes often outperforms some more sophisticated classification methods [26]. To demonstrate the Naïve Bayes Classification, for example, consider the $i_{th}$ example **p** which composed of *n* features $\left(f_{i,1},f_{i,2},\ldots ,f_{i,n}\right),f_{i,j}\in \left\{0,1,2\right\},j=1,2,3,\ldots ,n$, the Naïve Bayes algorithm predicts the class of **p** (denoted by $\hat{y}$) as that

(5) $$\hat{y}={\mathrm{argmax}}_{\mu }\left(P\left(y=\mu |X\right)\right)={\mathrm{argmax}}_{\mu }\left(\frac{P\left(X|y=\mu \right)P\left(y=\mu \right)}{P\left(X\right)}\right)\propto {\mathrm{argmax}}_{\mu }(P(X|y=\mu )P\left(y=\mu \right))$$

Multinomial Naïve Bayes assumes that each P(x|y=μ) is a multinomial distribution, so that

(6) $$P(X|y=\mu )=\overset{n}{\underset{i=1}{\prod }}P(x_{i}|y=\mu )=\overset{n}{\underset{i=1}{\prod }}\frac{n_{j}\left(\mu \right)+\alpha }{n_{j}+n\alpha }$$

where $n_{j}\left(\mu \right)$ denotes the sample number of the $j_{th}$ feature in the $\mu_{th}$ class.

### 4.4. Evaluation Measurements

To test the robustness of our method, we repeat the process of random selection of the training and test sets, model-building and model-evaluating on four parameters: overall prediction accuracy (Acc), sensitivity (Sn), specificity (Sp), Matthew’s correlation coefficient (MCC) [27,28,29,30,31,32,33] which would help us in determining how well the model would be generalized to new datasets. These parameters are defined as follows:

(7) $$\{\begin{array}{ll} Sn & =\frac{TP}{TP+FN}\times 100\%\\ Sp & =\frac{TN}{TN+FP}\times 100\%\\ Acc & =\frac{TP+TN}{TP+FN+TN+FP}\times 100\%\\ MCC & =\frac{TP*TN-FP*FN}{\sqrt{\left(TP+FP\right)\left(TP+FN\right)\left(TN+FP\right)\left(TN+FN\right)}}\times 100\% \end{array}$$

where TP, TN, FP and FN represent true positive, true negative, false positive and false negative, respectively. In our experiment, the Acc is the proportion of true results (the percentage of correctly identified bacteriophage virion and non-virion protein) among the total number of samples. The Sn is the proportion of bacteriophage virion protein that were correctly identified. The Sp measures the proportion of non-virion bacteriophage protein that was correctly identified. The MCC is a more stringent measure of taking into account true and false positives and negatives. In addition, it is a correlation coefficient between the observed and predicted binary classifications. The MCC returns a value in [−1, +1]. A coefficient of −1 indicates the disagreement between prediction and real facts, 0 is nearly random prediction, and +1 represents a perfect prediction. To depict the tradeoff between sensitivity and specificity (any increase in sensitivity will be accompanied by a decrease in specificity), the receiver operating characteristic (ROC) curves are also provided. The area under the curve is a measure of discrimination, that is, the ability of the test to correctly classify the bacteriophage virion proteins.

## 5. Web Server

User-friendly and publicly accessible web-servers [34,35,36,37,38,39,40,41,42,43,44] or databases [45,46,47] represent the future direction for developing practically more useful tools. Thus, a user-friendly web server called PhagePred was constructed for our work. Users may access the web server at http://bigroup.uestc.edu.cn/bacteriophage. The input of the web server is a set of protein sequences, which can either be uploaded as a single file or copied/pasted into the input box. Note that the input protein sequence should be in the FASTA format. The FASTA format sequence consists of a single initial line beginning with a greater-than symbol (“>”), followed by lines of amino acid sequence. After submitting the protein sequences and clicking the submit button, the predicted results will be shown in a new interface. For example, if you use the query sequences in the Example window as the input, you will see the following outcome on the screen: the 1st query example is identified as “bacteriophage virion” with probability 1; the 2nd query sample is identified as “bacteriophage non-virion” with probability 1. All these results are fully consistent with the experimental observations.

## 6. Conclusions

The application of bacteriophage virion proteins has wide medical and commercial value, which explains the interest in the identification of novel bacteriophage virion proteins. In fact, prediction of bacteriophage virion proteins does not only help in the discovery of many still unknown functions of bacteriophage but also in facilitating the design of new commercial and medical applications. Though some researchers have focused on this problem, the accuracy of prediction is still not satisfied. In this study, a Multinomial Naïve Bayes based approach was applied to the prediction of bacteriophage virion proteins by using sequence derived properties. The features generated from the g-gap feature tree contain more functional motifs than other methods, and they could better characterize the properties of bacteriophage virion proteins. Multinomial Naïve Bayes often outperforms some more sophisticated classification methods since it can effectively solve the problem of overfitting according to the experimental results. The high prediction accuracy on the training and testing datasets show that PhagePred is potentially a useful tool for predicting bacteriophage virion proteins from primary sequence. Because of its simplicity, this approach can easily be extended to recognizing other specific functional properties and should be a useful tool for high-throughput and large-scale analysis of proteomic and genomic data. Map-reduce techniques may be considered in future works [48]. The PhagePred program and dataset is available at http://bigroup.uestc.edu.cn/bacteriophage.

## Figures and Tables

**Table 1:** Comparison of PhagePred with other classifiers.

Classifier	Sn (%)	Sp (%)	Acc (%)	MCC (%)
xgboost	52.52	81.25	71.98	46.05
Random Forest	25.25	97.60	74.26	38.67
Adaboost + CART	52.53	88.94	77.20	41.03
SVM	73.74	90.87	85.34	65.92
**PhagePred**	**96.97**	**99.04**	**98.37**	**96.27**

Acc: accuracy, Sn: sensitivity, Sp: specificity, MCC: Matthew’s correlation coefficient, CART: Classification and Regression Trees.

**Table 2:** Comparison of state-of-the-art methods with PhagePred.

Classifier	Sn (%)	Sp (%)	Acc (%)	MCC (%)
Naïve bayes	75.76	80.77	79.15	54.59
SVM	75.76	89.42	85.02	65.53
PVP-SVM	73.73	93.27	86.97	69.50
**PhagePred**	**96.97**	**98.56**	**98.05**	**96.27**

Acc: accuracy, Sn: sensitivity, Sp: specificity, MCC: Matthew’s correlation coefficient.
